# Development and external validation of a perioperative clinical model for predicting myocardial injury after major abdominal surgery: A retrospective cohort study

**DOI:** 10.1016/j.heliyon.2024.e30940

**Published:** 2024-05-09

**Authors:** Guifen Fan, Hanjin Lai, Xiwen Wang, Yulu Feng, Zhongming Cao, Yuxin Qiu, Shihong Wen

**Affiliations:** a Department of Anesthesiology, The First Affiliated Hospital, Sun Yat-sen University, Guangzhou, China; b Department of Anesthesiology, Guangdong Provincial People's Hospital (Guangdong Academy of Medical Sciences), Southern Medical University, Guangzhou, China

**Keywords:** Myocardial injury, Major abdominal surgery, Troponin elevation, Predictive model

## Abstract

**Purpose:**

We aimed to develop and validate a predictive model for myocardial injury in individuals undergoing major abdominal surgery.

**Methods:**

This multicenter retrospective cohort analysis included 3546 patients aged ≥45 years who underwent major abdominal surgeries at two Chinese tertiary hospitals. The primary outcome was myocardial injury after noncardiac surgery (MINS), defined as prognostically relevant myocardial injury due to ischemia that occurs during or within 30 days after noncardiac surgery. The LASSO algorithm and logistic regression were used to construct a predictive model for postoperative MINS in the development cohort, and the performance of this prediction model was validated in an external independent cohort.

**Results:**

A total of 3546 patients were included in our study. MINS manifested in 338 (9.53 %) patients after surgery. The definitive predictive model for MINS was developed by incorporating age, American Society of Anesthesiologists (ASA) classification, preoperative hemoglobin concentration, preoperative serum ALB concentration, blood loss, total infusion volume, and operation time. The area under the curve (AUC) of our model was 0.838 and 0.821 in the derivation and validation cohorts, respectively.

**Conclusions:**

Preoperative hemoglobin levels, preoperative serum ALB concentrations, infusion volume, and blood loss are independent predictors of MINS. Our predictive model can prove valuable in identifying patients at moderate-to-high risk prior to non-cardiac surgery.

## 1 Introduction

Abdominal surgeries often involve extensive tissue manipulation and have unique perioperative challenges, including alterations in hemodynamics and potential impacts on cardiac function. Despite advances in surgical techniques, anesthetic agents, anesthesia management, and postoperative care, successful recuperation following major abdominal surgery is frequently impeded by the emergence of postoperative complications. The prevalence of complications still ranges from 11 % to 28 % following high-risk abdominal procedures [1].

Myocardial injury after non-cardiac surgery (MINS) is a prevalent complication among non-cardiac surgical patients and significantly contributes to perioperative morbidity and mortality [2]. Previous study found that the incidence of MINS was 43.8 % in patients undergoing open vascular surgeries involving the abdominal aorta surgery and had a significantly higher 30-day mortality rate compared to those who did not (12.0 % vs. 2.3 %) [3]. Postoperative myocardial injury was also shown to be associated with an increased risk of 1-year all-cause but not cardiac mortality [4]. In a substantial prospective cohort study, 7.9 % of patients were identified to have experienced MINS, 58.2 % of whom lacked ischemic features and consequently did not meet the universal definition of myocardial infarction. Patients who experienced MINS exhibited a significantly higher 30-day mortality rate compared to those who did not (9.8 % vs. 1.1 %) [5]. Moreover, the incidence of the composite outcome, including nonfatal cardiac arrest, nonfatal congestive heart failure, nonfatal stroke, and mortality, was notably more frequent among patients who suffered MINS. Another study demonstrated that 15.8 % of patients with MINS exhibited ischemic symptoms. Nevertheless, the long-term mortality of patients without ischemic symptoms did not differ from that of patients with ischemic symptoms [6,7]. Despite the extensive evaluation of the effectiveness of different preoperative interventions, including aspirin, α-2 agonists, β-blockers, and nitrous oxide, in reducing cardiovascular risk through large clinical trials, there is currently no widely acknowledged and safe approach proven to be effective in preventing perioperative myocardial infarction [[8], [9], [10]]. Given the hemodynamic changes induced by abdominal surgeries and their potential impact on cardiac function, it is essential for clinicians to utilize accurate risk stratification tools, which help identify patients at high risk of myocardial injury, thereby facilitating timely interventions and the optimization of perioperative management strategies. Previously reported predictive model was constructed primarily using preoperative factors such as age, methods of surgery, and American Society of Anesthesiologists (ASA) physical status [11]. We hypothesize that intraoperative factors may be associated with MINS and could provide valuable insights for predicting MINS. Despite attempts to create models for MINS prediction, these models have not achieved broad generalizability, largely due to a lack of external validation.

In our study, we retrospectively analyzed extensive pre- and intraoperative data from a substantial cohort. Subsequently, we developed and externally validated a novel predictive model to assess the risk of postoperative MINS following major abdominal procedures. The primary objective of this model is to function as a practical clinical tool for identifying individuals at increased risk of MINS. This approach allows timely intervention and supports personalized perioperative decision-making in the realm of major abdominal surgeries.

## 2 Methods

This study was guided by the Strengthening the Reporting of Observational Studies in Epidemiology (STROBE) guidelines [12] and the Transparent Reporting of a Multivariable Prediction Model for Individual Prognosis or Diagnosis (TRIPOD) reporting guidelines. Following these established frameworks ensures a comprehensive and transparent presentation of our observational study and predictive model, promoting the reliability and interpretability of our findings.

### 2.1 Study design and population

This study was approved by the Ethics Committee of The First Affiliated Hospital to Sun Yat-Sen University (Ethics approval reference FAHSYSU-2022-015). Considering the retrospective nature of this observational study, the requirement for written informed consent was waived.

We included consecutive patients who underwent major abdominal surgeries under general anesthesia at the First Affiliated Hospital of Sun Yat-sen University (Guangzhou, China) between January 2016 and December 2021. The exclusion criteria were as follows: (1) younger than 45 years of age; (2) not undergo high-sensitivity cardiac troponin detection preoperative or postoperative; (3) had a preoperative history of myocardial infarction, stroke, or infection diagnosed by clinicians or had preoperative high-sensitivity troponin T (hsTnT) levels exceeding 20 ng/L due to identifiable ischemic causes; and (4) lacked recorded weight and (or) height, amounts of blood loss, and infusion volume. For external validation, an independent cohort was assembled consisting of consecutive patients who underwent major abdominal surgery at the Guangdong Provincial People's Hospital (Guangzhou, China) between June 2019 and June 2022. The same exclusion criteria were applied.

### 2.2 Candidate predictors

Demographic and clinical data were extracted from electronic medical records, and the data collectors were blinded to our study's hypotheses to minimize potential bias.

The Candidate predictors included the following preoperative factors: age, sex, body mass index (BMI), ASA classification, history of hyperlipidemia, hypertension, chronic heart failure (CHF), coronary heart disease (CHD), chronic renal failure (CRF), chronic obstructive pulmonary disease (COPD), diabetes, anemia, hemoglobin levels, baseline platelet (PLT), hematocrit, preoperative serum albumin levels, estimated glomerular filtration rate (eGFR), and serum creatinine.

Intraoperative factors included the surgical method, duration of anesthesia, surgical time, total infusion volume, blood loss, intraoperative urine output, intraoperative hypotension (IOH), and methods of surgery, including laparotomy and minimally-invasive procedures.

Noninvasive blood pressure measurements were automatically recorded every 5 min within this system, allowing for the automatic calculation of mean arterial pressure. IOH was defined as a mean arterial pressure (MAP) below absolute thresholds of 65 mmHg for a cumulative duration of 5 min [13]. To further explore the relationship between hypotension and outcomes, we also included the cases where the mean arterial pressure fell below 60 mmHg for a cumulative duration of 5 min.

Additionally, details regarding the length of hospital stay and postoperative outcomes were meticulously documented. These comprehensive datasets formed the basis for our analyses, providing a thorough understanding of the patient profile and perioperative events.

### 2.3 Outcomes

The primary outcome of the study was MINS, which was defined as myocardial ischemia within 30 days after non-cardiac surgery according to the 2014 American College of Cardiology and American Heart Association (ACC/AHA). Regardless of whether clinical signs and symptoms of ischemia are present, the troponin assay-specific thresholds for the diagnosis of MINS that are associated with adverse prognosis are postoperative hsTnT between 20 and 65 ng/L with an absolute change ≥5 ng/L, any elevation ≥65 ng/L or any absolute change ≥14 ng/L [14]. The hsTnT level was used to diagnose myocardial injury in our cohort, and the most recent hsTnT level before surgery was considered as the baseline. In our study, Troponin assays utilized in this study were consistent with contemporary clinical practice standards. Blood samples for the 5th generation (high-sensitivity) troponin T were obtained preoperatively up to 2 weeks before surgery, and within the initial 2–3 days following surgery during the hospital stay [15]. Patients identified with specific risk factors for myocardial injury during preoperative assessment, such as advanced age, preexisting cardiovascular disease, or prolonged surgical duration, typically undergo routine blood sampling for cardiac troponin measurement. Blood samples for troponin measurement may also be drawn when patients experience symptoms such as dyspnea or pain in the chest, neck, or arms. The 5th generation troponin assay offers improved sensitivity and specificity compared to earlier generations, aligning with diagnostic standards in cardiac biomarker testing. All the data were extracted from the structural history data collection system. Other nonischemic or extracardiac causes leading to elevated hsTnT levels, such as sepsis, pulmonary embolism, chronic nonischemic cardiac troponin T (cTnT) elevation, rapid atrial fibrillation, or severe anemia, were excluded from the analysis.

### 2.4 Model development

Two computer algorithms were employed to select predictive factors in the development cohort. Initially, both univariate and multivariate logistic regression analyses were conducted to identify potential risk factors. Independent variables with P values < 0.05 in the univariate logistic regression analysis were subsequently included in the multivariate logistic regression analysis. To prevent overfitting, the variables were subjected to the least absolute shrinkage and selection operator (LASSO) algorithm, which selects the most essential potential predictors with nonzero coefficients. Next, a 10-fold cross-validation was executed to determine the optimal value of lambda (λ). To assess the performance of the two methods on the development cohort, we compared the area under the curve (AUC), Akaike information criterion (AIC), and Bayesian information criterion (BIC) (where smaller values indicate better performance).

Following LASSO regression, our initial model identified nine predictor variables associated with myocardial injury following major abdominal surgery including ASA classification, chronic renal failure, chronic obstructive pulmonary disease, surgical time, intraoperative blood loss, age, hemoglobin, albumin, and infusion volume. However, the decision to exclude "chronic renal failure" and "chronic obstructive pulmonary disease" from the final model was based on clinical expertise and considerations of redundancy and practicality in clinical practice. In clinical practice, physicians often incorporate comorbidities such as chronic renal failure and heart failure into the assessment of ASA classification, which is widely used for preoperative risk stratification. Given that these comorbidities are already accounted for in the ASA classification, their inclusion as separate predictor variables in the model may introduce redundancy. Therefore, we opted to exclude these variables from the final model to streamline its interpretability and enhance clinical applicability.

The final model underwent a rigorous validation process to ensure that all variables met the criteria for the variance inflation factor (VIF). We calculated the area under the receiver operating characteristic (ROC) curve (AUC) with a 95 % confidence interval (CI) to compare the performance of the predictive models. The calibration of the model was assessed by comparing its goodness-of-fit to the ideal calibration curve utilizing the calibration slope and Brier score.

Furthermore, we conducted decision curve analysis (DCA) to evaluate the clinical utility of the model by quantifying the net benefits at various threshold probabilities. Ultimately, the model was presented in the form of a nomogram created using R Studio.

### 2.5 Statistical analysis

For the establishment of the model, a ratio of the number of subjects to the number of candidate predictor parameters greater than 20 was considered reasonable for the number of subjects with specific clinical outcome events [16]. Considering a data collection loss rate of 10 %, this study needed to include a minimum sample size of approximately 712 patients. We analyzed all eligible patients to maximize the statistical power. Patients from the First Affiliated Hospital of Sun Yat-sen University composed the development cohort, while those from the Guangdong Provincial People's Hospital composed the validation cohort.

Before data analysis, predictor variables in the development and validation cohorts were inspected for missing values. Among the predictors, the proportion of missing preoperative laboratory values including hemoglobin, hematocrit, albumin, and platelet were less than 1.5 %. Multiple imputations by chained equations were used to impute missing data, using the mice package for R, in which predictive mean matching is embedded with the cases (k) = 5 default. Statistical analyses were performed using R statistical software version 4.2.0 (R Foundation for Statistical Computing). The raw data were processed using the Table package in R with Robust Multiarray Analysis (RMA). This study did not include data missing hsTnT values, and the remaining data were filled out using multiple imputation. Continuous variables are presented as medians (interquartile ranges (IQRs)) and were compared using Student's *t*-test. Categorical variables are presented as counts and percentages (%) and were compared using a chi-square test or Fisher's exact test, as appropriate.

The R package glm was utilized for logistic regression, and bootstrap methods were applied to test the robustness of the significant candidate factors chosen by the logistic model. The odds ratio (OR) and 95 % confidence interval (CI) of all baseline characteristics were calculated as a measure of linear association between clinical variables and MINS. Collinearity between variables was assessed based on the VIF. To evaluate the discrimination and calibration of the final prediction model, we constructed ROC curves and calibration curves and performed a Hosmer-Lemeshow test. Clinical effectiveness was further estimated through decision curve analysis.

In addition to formulating a nomogram, we developed a web application for conveniently inputting individual patient characteristics to predict the probability of MINS.

## 3 Results

### 3.1 Cohort characteristics

**Fig. 1:**
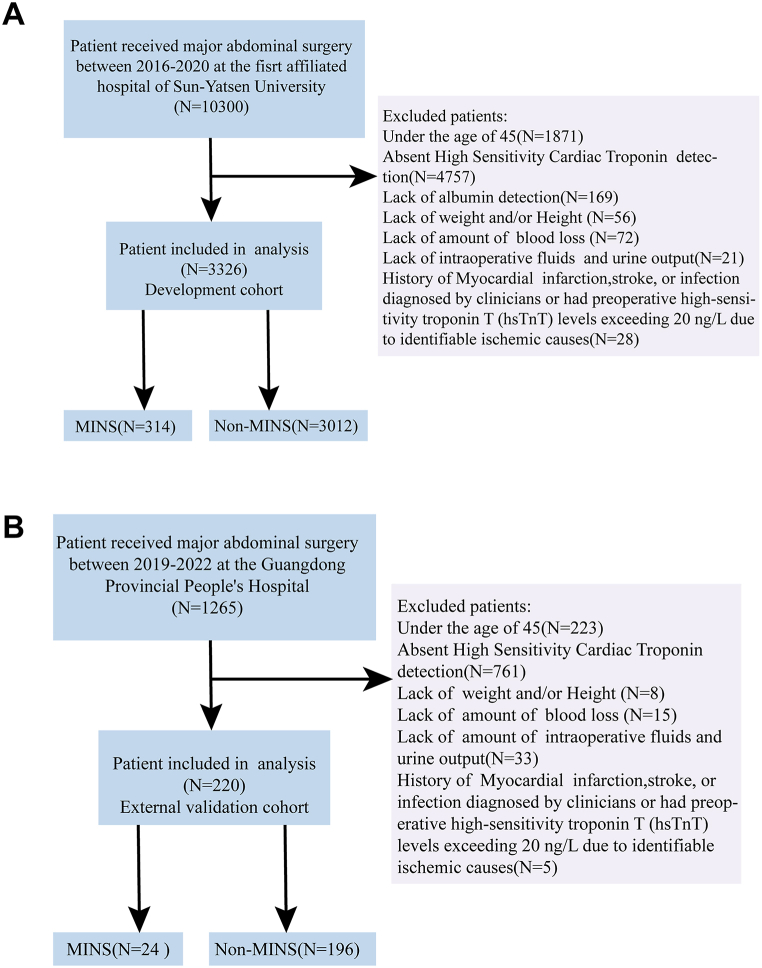
Flow diagram of the prediction model for the development (**A**) and external validation (**B**) cohorts.

In the development cohort, out of 10,300 participants who underwent major abdominal surgeries during the study period. After exclusion criteria, 3326 patients were finally analyzed. As demonstrated, the incidence of MINS was 9.4 % (314/3326) in the development cohort (Fig. 1A). The mean (SD) age was 61 years, and 67.3 % were male. The surgical method employed was laparotomy in 84.4 % of the patients, with minimally invasive procedures (15.6 %, Table 1). For the validation cohort, 220 individuals met the inclusion criteria and were enrolled in the study. The incidence of MINS was 10.4 % (24/220) in the validation cohort (Fig. 1B). The mean (SD) age was 66.5 years, with 62.3 % of participants being male. Laparotomy was the surgical method for 27.7 % of patients, while minimally invasive procedures accounted for 72.3 %. In the development cohort, the most prevalent comorbidities included anemia (55.5 %), hypertension (39.5 %), and diabetes (21.4 %, Table 1). Overall median age of the study population was 62 years, and 66.9 % were male. The most two predominant surgical procedures were colorectal resection (39.8 %) and gastrectomy (23 %). The distribution of ASA physical status was comparable in both cohorts, with ASA II being the most prevalent (69.3 %), followed by ASA III (23.4 %).

**Table 1:** Baseline characteristics of patients.

Variables	Total (n = 3546）	Development cohort (n = 3326)	External validation(n = 220)	*P* Value
Age, yr	62.00 [54.00, 69.00]	61.00 [54.00, 68.00]	66.50 [60.00, 74.25]	<0.001
Age <65 yr (%)	2153 (60.7)	2059 (61.9)	94 (42.7)	
Age ≥65 yr (%)	1393 (39.3)	1267 (38.1)	126 (57.3)	<0.001
**Sex (%)**				
Female	1172 (33.1)	1089 (32.7)	83 (37.7)	
Male	2374 (66.9)	2237 (67.3)	137 (62.3)	0.148
Body mass index (BMI), kg/m^2^	22.04 [19.94, 24.17]	22.04 [19.88, 24.16]	22.43 [20.58, 24.40]	0.015
**Methods of surgery**				
Laparotomy	2869 (80.9)	2808 (84.4)	61 (27.7)	<0.001
Minimally-invasive procedures	677 (19.1)	518 (15.6)	159 (72.3)	
**ASA classification (%)**				<0.001
I	141 (4.0)	131 (3.9)	10 (4.6)	
II	2457 (69.3)	2341 (70.4)	116 (53.0)	
III	829 (23.4)	759 (22.8)	70 (32.0)	
IV	118 (3.3)	95 (2.9)	23 (10.5)	
**Associated illness, No. (%)**				
Hypertension	763 (21.5)	676 (20.3)	87 (39.5)	<0.001
Anemia	1644 (46.4)	1522 (45.8)	122 (55.5)	0.006
Diabetes	417 (11.8)	370 (11.1)	47 (21.4)	<0.001
Coronary heart disease (CHD)	147 (4.1)	113 (3.4)	34 (15.5)	<0.001
Chronic renal failure (CRF)	44 (1.2)	36 (1.1)	8 (3.6)	0.003
Chronic heart failure (CHF)	28 (0.8)	22 (0.7)	6 (2.7)	0.003
Hyperlipidemia	194 (5.5)	184 (5.5)	10 (4.5)	0.638
Pre-operative laboratory findings				
Hemoglobin, g/L (median [IQR])	122.00 [103.00, 136.00]	123.00 [103.00, 137.00]	112.80 [98.50, 127.25]	<0.001
PLT, × 10^9^ (median [IQR])	215.00 [161.00, 282.00]	222.00 [169.25, 288.00]	113.00 [60.75, 165.25]	<0.001
Hematocrit (median [IQR])	0.37 [0.32, 0.41]	0.37 [0.32, 0.41]	0.34 [0.30, 0.39]	<0.001
Hematocrit <0.35	1350 (38.1)	1226 (36.9)	124 (56.4)	<0.001
Albumin, g/L (median [IQR])	36.40 [31.50, 39.60]	36.70 [32.70, 39.80]	27.80 [27.80, 27.80]	<0.001
Serum albumin <34 g/L	1057 (29.8)	1046 (31.4)	11 (5.0)	<0.001
eGFR >60 (ml/min/1.73 m)	3317 (93.5)	3103 (93.3)	214 (97.3)	0.029
Serum creatinine ≤176.8ummol/L	3508 (98.9)	3290 (98.9)	218 (99.1)	1
**Intraoperative characteristics**				
Duration of anesthesia, min (median [IQR])	315.00 [250.00, 400.00]	320.00 [250.00, 400.00]	300.00 [237.75, 388.50]	0.006
Surgical time, h (median [IQR])	4.32 [3.25, 5.67]	4.41 [3.32,5.75]	3.33 [2.38, 4.27]	<0.001
Surgical time > 3h (%)	2801 (79.0)	2669 (80.2)	132 (60.0)	<0.001
Blood loss, cc (median [IQR])	120.42 [0.00, 300.00]	100.00 [0.00, 300.00]	160.24 [0.00, 399.02]	0.395
Blood loss >20 cc/kg	196 (5.5)	193 (5.8)	3 (1.4)	0.008
Infusion volume, ml/h (median [IQR])	589.20 [471.60, 731.25]	115.80 [94.05, 167.40]	600.00 [490.80, 739.80]	<0.001
Number of patients with IOH (%)				
MAP <65 mmHg	3327 (93.8)	3111 (93.5)	216 (98.2)	0.009
MAP <60 mmHg	3227 (91.0)	3030 (91.1)	197 (89.5)	0.007
Intraoperative urine output, ml/kg/h (median [IQR])	1.65 [0.94, 2.88]	1.54 [0.91, 2.61]	5.40 [4.17, 6.50]	<0.001
**Top 10 surgical procedures, No. (%)**				<0.001
Gastrectomy	817 (23.0)	735 (22.1)	82 (37.3)	
Hepatectomy	757 (21.3)	747 (22.5)	10 (4.5)	
Colorectal resection	1411 (39.8)	1322 (39.7)	89 (40.5)	
Pancreatectomy	240 (6.8)	216 (6.5)	24 (10.9)	
Esophageal resection	131 (3.7)	131 (3.9)	0 (0.0)	
Exploratory laparotomy	113 (3.2)	113 (3.4)	0 (0.0)	
Cholecystostomy	47 (1.3)	47 (1.4)	0 (0.0)	
Other OR GI therapeutic procedures	30 (0.8)	15 (0.5)	15 (6.8)	
**Postoperative data**				
Length of stay, day (median [IQR])	18.00 [14.00, 27.00]	18.00 [14.00, 27.00]	16.00 [10.00, 23.25]	<0.001
Postoperative myocardial injury, n (%）	338 (9.5)	314 (9.4)	24 (10.9)	0.549
Postoperative acute kidney injury, n (%)	601 (16.9)	598 (18.0)	3 (1.4)	<0.001
Postoperative atelectasis, n (%)	535 (16.1)	534 (16.1)	1 (50.0)	0.731

Continuous data are reported as mean ± SD or median (25 % percentile, 75 % percentile). Categorical data are given as counts (percentages).

ASA = American Society of Anesthesiologists; eGFR = estimated glomerular filtration rate; MAP = mean arterial pressure; IOH = intraoperative hypotension; IQR = Interquartile Range.

### 3.2 Model development

**Fig. 2:**
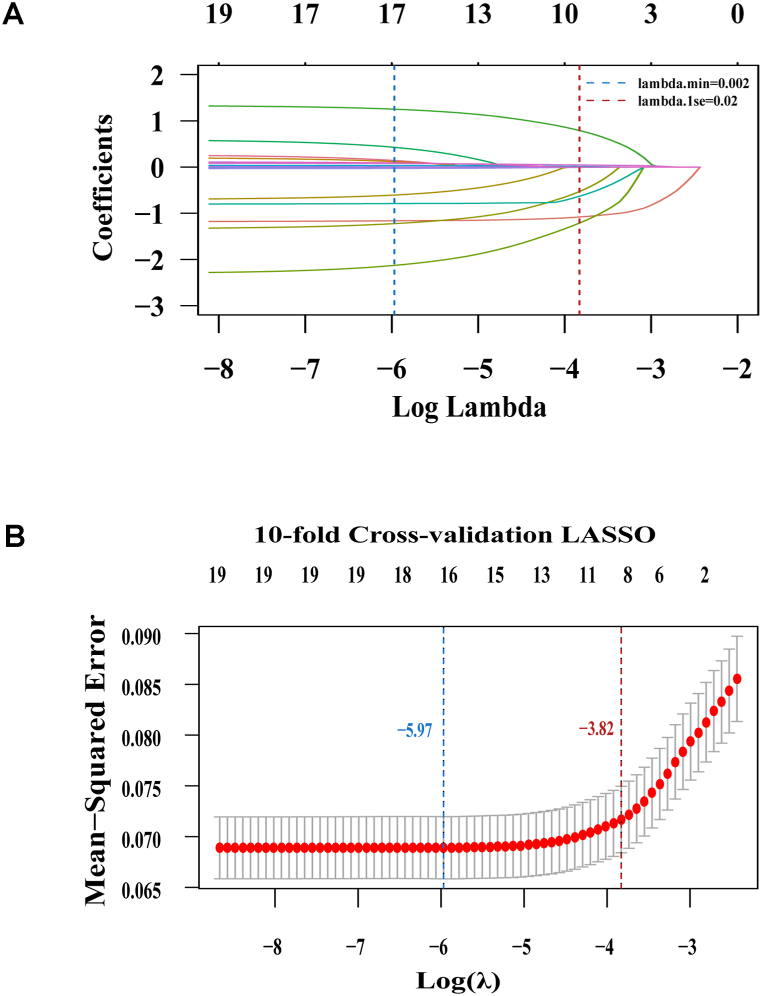
Feature variable selection using least absolute shrinkage and selection operator (LASSO) regression in the development cohort. **(A)** A LASSO coefficient profile depicting the relationship between all feature variables and the log (λ) sequence is presented. The blue and red dotted vertical lines represent the log (λmin) and log (λ1 se), respectively. Nine predictors with nonzero coefficients were identified based on the log (λ1 se) value. **(B)** LASSO regularization with 10-fold cross-validation was utilized to determine the optimal parameter (λ) based on the minimal mean squared error (MSE). The coefficients are plotted against log (λ). The two dotted vertical lines indicate the optimal λ values based on either the criterion of minimal MSE (λmin) or the criterion of one standard error of the minimum (λ1 se). The optimal values for log (λ1 se) and λ1 se were −5.97 and 0.02, respectively. (For interpretation of the references to color in this figure legend, the reader is referred to the Web version of this article.)

Two computer algorithms were used to select predictive factors in the development cohort. We first performed univariate logistic regression analysis to screen potential risk factors in the development cohort. Nineteen independent variables with P values < 0.05 in univariate analysis were selected for multivariable logistic regression analysis. As shown in Table 2, 12 candidate predictors remained significant after both forward and backward stepwise multivariate logistic regression analysis. To avoid overfitting of the model, we performed LASSO algorithm to select the most essential potential predictors with nonzero coefficients (Fig. 2A). A similar logistic regression model was constructed, but the variables were retained using more stringent thresholds, thus helping to select a parsimonious, predictive subset of variables. Then, we performed a 10-fold cross-validation to determine the optimal value of lambda (λ). Finally, nine candidate predictors were retained in this model when λ increased to 0.02 (one standard error of the minimum λ); these were potentially the most influential variables for predicting MINS (Fig. 2B).

**Table 2:** Univariable and multivariable analysis.

Variables	Univariable analysis		Multivariable analysis	
	Odds ratio (95%CI)	P value	Odds ratio (95%CI)	P value
Age	1.06 (1.05–1.08)	<0.001	1.04 (1.02–1.06)	<0.001
Male sex	1.19 (0.93–1.54)	0.1724		
Body mass index（BMI), kg/m^2^	0.93 (0.9–0.97)	<0.001	0.97 (0.93–1.01)	0.119
Methods of surgery				
Minimally-invasive procedures	Reference			
Laparotomy	1.76 (1.2–2.59)	0.0038	1.39 (0.89–2.17)	0.1475
ASA classification				
I–II	Reference		Reference	
III	5.18 (3.97–6.76)	<0.001	2.93 (2.14–4.01)	<0.001
IV	48.36 (30.1–77.71)	<0.001	17.95 (10.15–31.76)	<0.001
Anemia	2.95 (2.25–3.85)	<0.001		
Hyperlipidemia	1.55 (1–2.41)	0.05		
Hypertension	1.72 (1.33–2.23)	<0.001		
Chronic heart failure (CHF)	21.54 (8.71–53.26)	<0.001	7.52 (2.44–23.21)	<0.001
Coronary heart disease (CHD)	3.55 (2.29–5.5)	<0.001	2.37 (1.38–4.08)	0.0017
Chronic renal failure (CRF)	10.11 (5.2–19.66)	<0.001	4.41 (1.98–9.81)	<0.001
Chronic obstructive pulmonary disease (COPD)	5.12 (3.72–7.05)	<0.001	4.07 (2.80–5.98)	<0.001
Diabetes	1.82 (1.33–2.49)	<0.001		
Hemoglobin, g/L	0.97 (0.97–0.98)	<0.001	0.99 (0.98–1.00)	0.002
Albumin, g/L	0.92 (0.9–0.94)	<0.001	0.97 (0.94–0.99)	0.008
Surgical time, h	1.09 (1.04–1.13)	<0.001	1.15 (1.08–1.22)	<0.001
Total Infusion volume, L (median [IQR])	1.21 (1.16–1.27)	<0.001	1.06 (1–1.13)	<0.001
Blood loss >20 cc/kg	5.26 (3.78–7.31)	<0.001	2.12 (1.29–3.48)	0.003
Intraoperative urine output, ml/kg/h	1.06 (1.01–1.11)	0.0236		
IOH (MAP <65 mmHg)	0.82 (0.53–1.27)	0.3727		
IOH (MAP <60 mmHg)	0.88 (0.6–1.3)	0.5248		
Surgical procedures				
Gastrectomy	Reference		Reference	
Cholecystostomy	0.94 (0.33–2.71)	0.9131		
Colorectal resection	1.14 (0.84–1.56)	0.3965		
Esophageal resection	0.75 (0.36–1.54)	0.4305		
Exploratory laparotomy	2.73 (1.63–4.59)	<0.001	3.7 (1.92–7.12)	0.106
Hepatectomy	0.74 (0.51–1.09)	0.1256		
Other OR GI therapeutic procedures	5.07 (1.68–15.28)	0.0039	2.03 (0.59–7.03)	0.262
Pancreatectomy	1.09 (0.65–1.83)	0.7393		

ASA = American Society of Anesthesiologists; MAP = mean arterial pressure; IOH = intraoperative hypotension; IQR=Interquartile Range.

The performance of the two methods against the development cohort is shown in Table 3. The AUC, AIC and BIC were compared. Finally, seven variables, namely, ASA grade, blood loss volume (≤20, >20 mL/kg), age, preoperative hemoglobin (Hemoglobin), preoperative serum ALB concentration (ALB), intraoperative infusion volume and operation time, may be independently related to the occurrence of MINS.

**Table 3:** Performance of feature variable selection method in the development cohort.

Feature Selection Method	No. of variables	AUC (95%CI)	AIC	BIC
Univariate and multivariate logistic stepwise regression	12	0.860 (0.838–0.883)	1522.115	1644.306
A lasso regularization	9	0.849 (0.825–0.872)	1543.178	1610.383

AUC = area under the curve; AIC = Akaike information criterion; BIC = Bayesian information criterion; CI = confidence interval; No. = number.

### 3.3 Model evaluation

**Fig. 3:**
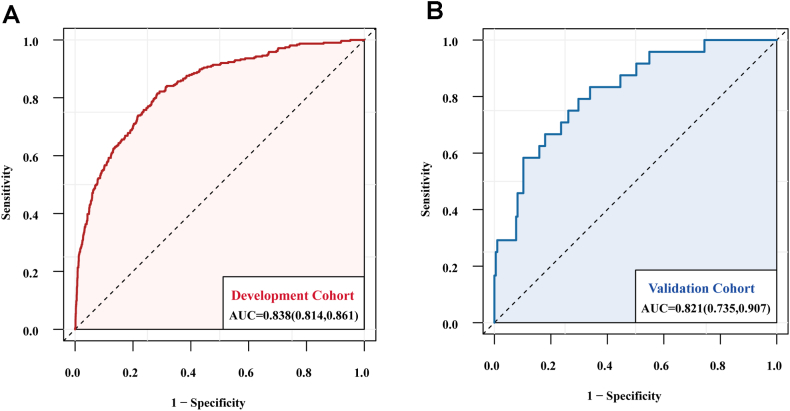
ROC curve for the prediction model in the development (**A**) and external validation (**B**) cohorts.

**Fig. 4:**
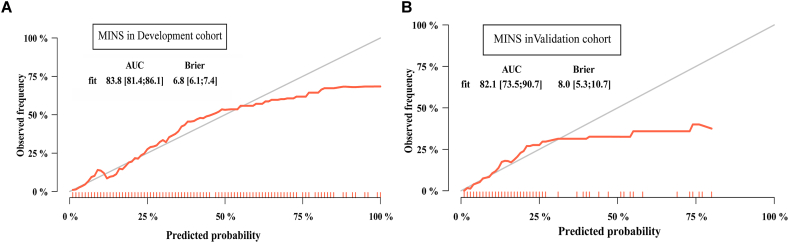
Calibration curves for testing the stability of the prediction model in the development (**A**) and external validation (**B**) cohorts. Red logistic calibration curves are shown, and gray lines represent the ideal reference lines. The statistical results are displayed in the upper left corner of each plot. (For interpretation of the references to color in this figure legend, the reader is referred to the Web version of this article.)

The final predictors were age, ASA grade, Hemoglobin, ALB, blood loss during surgery, total infusion volume and duration of surgery. The discriminatory ability of our prediction model was assessed by plotting the area under the ROC curve (AUC), which is an intuitive way to assess the clinical utility of diagnostic and predictive models. The final prediction model showed excellent discrimination performance. The apparent AUC of the MINS prediction model in the development cohort was 0.838 (95 % CI, 0.814–0.861; Fig. 3A). Moreover, the AUC in the external validation cohort was 0.821 (95 % CI, 0.735–0.907; Fig. 3B). The calibration plots of both cohorts were almost diagonal, and the results of the Hosmer-Lemeshow goodness-of-fit test showed no statistical significance (training cohort: chi-square = 9.46, *P* = 0.396; validation cohort: chi-square = 10.19, *P* = 0.335), indicating that this model was well calibrated with good agreement between the observed and predicted risk. As shown, the Brier score was 0.068 in the development cohort (Fig. 4A) and 0.080 in the validation cohort (Fig. 4B).

**Fig. 5:**
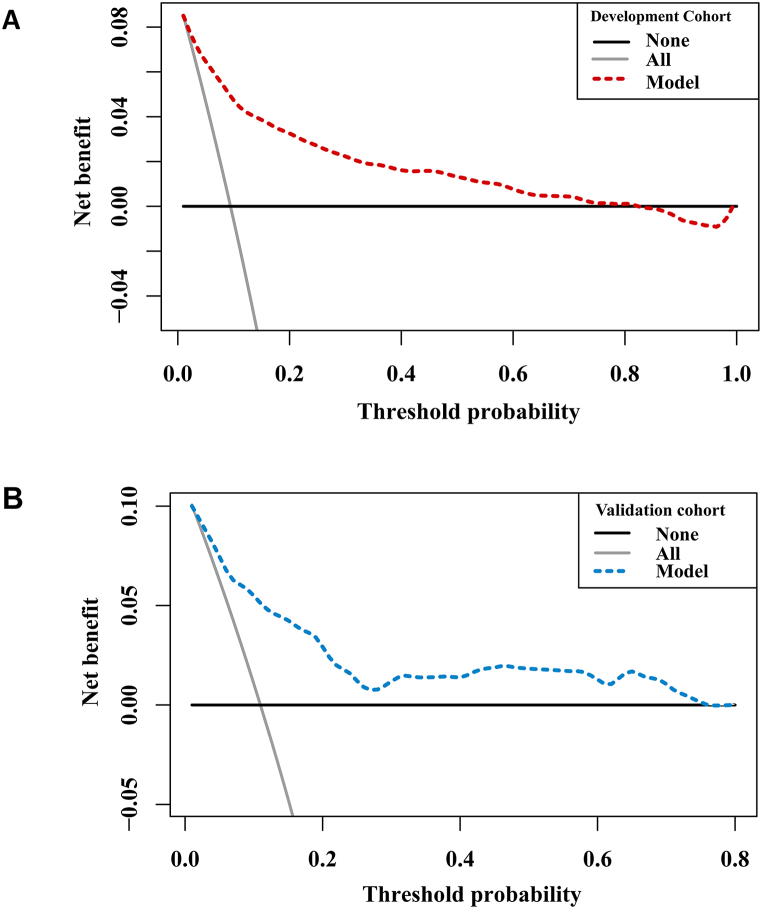
Decision curve analysis of the prediction model for the development (**A**) and external validation (**B**) cohorts. The net benefits (dashed lines) of the prediction model are presented as a function of the sequential probability thresholds for MINS. The gray and black lines indicate the hypothetical conditions of all patients who underwent MINS or not. The prediction model demonstrated satisfactory clinical significance, with constantly positive net benefits across a wide range of decision thresholds.

To evaluate the clinical utility of the prediction model across different threshold probabilities, we conducted a DCA to quantify the net benefits. DCA is a statistical method used to evaluate the clinical utility of predictive models or diagnostic tests by assessing their net benefit across a range of decision thresholds [17]. It quantifies the clinical value of a model by comparing the benefit of using the model to guide decision-making against the harm of both treating and not treating patients. In DCA, the net benefit is quantitatively expressed as the difference between the proportion of true positives (sensitivity) and a weighted proportion of false positives (1-specificity), where the weights reflect the relative harm of false-positive and false-negative predictions [18]. A positive net benefit indicates that using the model to guide decision-making yields more benefit than harm, while a negative net benefit suggests that the model's use may lead to more harm than benefit. With respect to the development cohort, the model demonstrated superior net benefits when clinical decision thresholds fell within the range of 0 %–70 % of the predicted risk, outperforming both the treat-all-patient and treat-no-patient schemes (Fig. 5A). With respect to the external validation cohort, the corresponding threshold probabilities ranged from 2 % to 100 % (Fig. 5B).

**Fig. 6:**
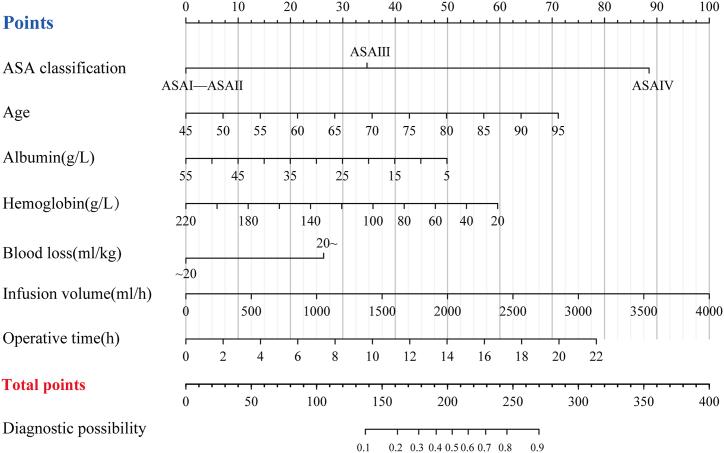
Nomogram of the predictive model for the risk of MINS.

These findings contribute valuable insights into the role of preoperative clinical parameters in optimizing the risk assessment of MINS in patients undergoing major abdominal surgery. The final prediction model was presented as a nomogram (Fig. 6). The score for each predictor was determined by drawing a vertical line from the predictor line to the points line. The cumulative points score, obtained by summing the points from all the predictors, corresponds to the predicted probability of MINS. To enhance the accessibility and convenience of this model, we developed a web page for risk prediction (https://minsprediction.shinyapps.io/MINSprediction). For example, a patient was 70 years old and classified as ASA III, with a baseline preoperative albumin level of 30 and a hemoglobin level of 100; blood loss of 20 ml/kg; intraoperative fluid volume of 1504 ml/h; and an operation time of 9 h. His risk of developing MINS was 0.572 (95 % CI, 0.684–0.778).

## 4 Discussion

In our present investigation, we identified seven independent predictors associated with MINS following major abdominal procedures. These predictors included age, ASA classification, Hemoglobin levels, ALB levels, intraoperative blood loss, total infusion volume, and duration of surgery. Leveraging these identified predictors, we constructed a nomogram specifically tailored for MINS risk assessment, allowing for the stratification of patients based on their estimated risk of developing MINS. This nomogram serves as a valuable tool in clinical decision-making for optimizing patient care and intervention strategies in the context of major abdominal surgeries.

Myocardial injury is a prevalent occurrence in patients undergoing non-cardiac surgery and is significantly correlated with long-term mortality [15]. Annually, approximately 200 million adults globally undergo major non-cardiac surgical procedures [19]. Within this population, an estimated 8 million individuals develop MINS [20], an event independently associated with an increased risk of death and cardiovascular complications during the initial postoperative year [21]. The perioperative mortality following non-cardiac surgery is 1%–2% among inpatients aged ≥45 years, with half of these fatalities attributed to cardiovascular complications stemming from surgical procedures [2]. The Vascular Events In Noncardiac Surgery Patients Cohort Evaluation (VISION) study published the largest cohort of non-cardiac surgical patients screened for myocardial injury that 8.0 % of individuals aged 45 years or older who were undergoing non-cardiac surgeries experienced MINS [5]. Similarly, within our cohorts comprising 3546 patients undergoing major abdominal surgery, we observed an overall incidence of MINS of 9.53 %. Concurrently, the average hospital length of stay for patients who underwent MINS (34 days) exceeded that of patients in the non-MINS group (21 days).

VISION investigators have identified several preoperative predictors of MINS, including age (75 years or older), cardiovascular risk factors (known as cardiovascular diseases), and surgical factors (urgent/emergent surgery) [5]. While the Revised Cardiac Risk Index is commonly used for cardiac risk stratification, its discriminative ability is relatively low [22]. In addition, intraoperative hemodynamics have been found to influence the risk of MINS, and the surgical Apgar score (SAS) was developed to assess these risk factors [23]. However, in clinical practice, the assessment of these risk factors can be intricate and may necessitate additional diagnostic tests, posing challenges for accurate prediction. Furthermore, existing tools for predicting MINS often rely on preoperative variables, which can be challenging to standardize rapidly, particularly in emergency surgical scenarios. Despite these challenges, a predictive model for MINS was developed by Ana Belen Serrano in 2020 [11]; however, these results may not be generalizable to other settings because they include only nonmodifiable predictive factors and were generated at a single center. Furthermore, when applied to our development dataset, the model exhibited a comparatively lower C-statistic of 0.697. Therefore, our study presents a more practical and effective predictive model for clinicians to assess the risk of MINS and identify patients at high risk of developing myocardial ischemia. Our model demonstrated satisfactory discrimination and calibration, with consistent performance in both the development and external validation datasets (AUCs of 0.84 and 0.82, respectively). The positive net benefit of our model does not directly impact patient prognosis but reflects its ability to provide information for clinical decision-making and resource allocation more effectively, ultimately improving patient care and optimizing healthcare resources. Essentially, DCA helps clinicians determine whether the use of predictive models can lead to better patient outcomes. For instance, our model may help clinicians identify high-risk patients for postoperative myocardial ischemia early after major abdominal surgery and tailor monitoring, prevention, and treatment strategies accordingly, or admit high-risk patients to the ICU to improve patient outcomes. Besides, one of the purposes of this study is to find the risk factors of MINS after major abdominal surgery. When patients with major abdominal surgery have the risk factors screened out in this study, a routine troponin detection is recommended to identify and intervene with MINS as early as possible.

To the best of our knowledge, this is the first externally validated MINS prediction model. We have developed a straightforward and visually intuitive prediction model that can serve as a valuable guide for medical professionals.

The ASA classification is a globally employed tool for evaluating the preoperative health status of patients [24]. Recent studies have substantiated its robustness as a standalone predictor of postoperative complications and mortality [25]. Our findings also highlight the ASA classification as a robust and independent predictor of postoperative MINS in patients who underwent major abdominal surgery.

A large-scale study reported an independent association between preoperative anemia and MINS, suggesting a potential correlation between anemia and postoperative mortality [26]. Another prospective clinical study identified preoperative anemia as an independent risk factor for increased cardiovascular events during the perioperative period in vascular surgery patients [27]. However, in previous studies, anemia was not included in various risk prediction models for myocardial injury. In our research, we found that preoperative hemoglobin levels are independently associated with myocardial injury after non-cardiac surgery. The hemoglobin concentration serves as a crucial indicator of the blood's capacity for oxygenation. Insufficient delivery of oxygen to the myocardium has the potential to induce contractile dysfunction, impacting the heart's ability to effectively pump blood and compromise overall cardiac function [28].

Albumin is one of the primary proteins in plasma and functions by maintaining osmotic pressure, transporting nutrients and hormones, and regulating acid‒base balance [29]. It was found that lower preoperative serum ALB concentrations are associated with an increased risk of coronary disease and all-cause mortality [30]. Potential causes may include hypoalbuminemia inducing interstitial fluid influx due to decreased blood vessel osmolarity, resulting in diminished blood volume [29]. This circumstance may necessitate intensified cardiac pumping efforts to sustain adequate perfusion. Moreover, hypoalbuminemia may be linked to a chronic inflammatory state, where alterations in immune responses could impact the well-being of myocardial cells [31]. However, the specific mechanisms leading to CVD still require further research.

A large-scale prospective clinical study reported a correlation between substantial perioperative blood loss in non-cardiac surgery patients and the occurrence of postoperative myocardial infarction [32]. In our investigation, we observed a heightened susceptibility to ischemic complications among patients who underwent major abdominal surgery, particularly when their blood loss exceeded 20 ml/kg (OR, 2.12; 95 % CI, 1.29 to 3.48; *P* = 0.003). This emphasizes the need for vigilant perioperative care. To reduce the risk of MINS after major abdominal surgery, medical staff should avoid fluid overload in patients at high risk of MINS during surgery and try to shorten the surgery time as much as possible.

In our study, we did not observe an association between hypotension and outcomes, which may be attributed to the specific thresholds and durations of hypotension selected for analysis. Future research may warrant a reassessment of hypotension definitions to ascertain the most predictive and clinically significant blood pressure thresholds and durations. While chronic renal failure, COPD, and CHF are associated with an increased risk of postoperative myocardial injury, we exclude these factors from our final predictive model. This decision was based on several considerations. Firstly, it is important to note that the ASA classification system, which is a key component of our predictive model, already incorporates these comorbidities to some extent. The ASA classification system considers a wide range of patient factors, including chronic medical conditions, in assessing the overall perioperative risk. Therefore, the inclusion of chronic renal failure, COPD, and CHF as separate variables in our model would result in redundancy and unnecessary complexity. Secondly, during our model development process, we found that the individual impact of chronic renal failure, COPD, and CHF on the predictive efficacy of the model was relatively small compared to other variables. While these comorbidities certainly contribute to the overall risk profile of patients undergoing abdominal surgery, their exclusion from the final model did not significantly compromise the model's ability to accurately predict postoperative myocardial injury. In the interest of practicality and ease of use in clinical settings, we opted to focus on the most influential and clinically relevant variables in our predictive model.

Identifying patients at high risk of developing MINS is crucial for timely intervention and prevention of adverse outcomes. Elevated troponin levels, indicative of myocardial injury, have been widely recognized as a prognostic marker for adverse outcomes following various clinical scenarios, including major abdominal surgery [33]. Several studies have demonstrated a clear correlation between elevated troponin levels and mortality across diverse patient populations and clinical settings. Grant et al. conducted an observational study involving 12,882 patients undergoing noncardiac vascular surgery and found a significant association between postoperative troponin elevation and increased long-term mortality [34]. Another prospective observational cohort study found postoperative high-sensitive cardiac troponin T was a strong predictor of non-cardiac 30 day complications, increased hospital stay and hospital mortality in patients undergoing major abdominal surgery [1]. The 10.13039/100013500Canadian Cardiovascular Society Guidelines on Perioperative Cardiac Risk Assessment and Management recommend obtaining daily troponin measurements for 48–72 h after surgery in patients with a baseline risk of greater than 5 % for cardiovascular deaths or nonfatal myocardial infarction at 30 days after surgery [35]. The elevation of preoperative hs-cTnT concentration is significantly associated with long-term mortality after noncardiac surgery, one-third of which may be attributable to MINS [36]. However, in clinical practice, hsTnT assays are often performed only for patients with symptoms or a known history of CVD during hospitalization [37]. In our study, 46 % of patients were excluded due to a lack of postoperative hsTnT measurements. While the cost of monitoring troponin levels may be a concern, it is important to note that the cost of treating adverse cardiac events can be much greater [38]. Postoperative diagnosis of MINS is always neglected in patients after major abdominal surgery because symptoms such as chest pain or electrocardiogram changes are usually delayed [39]. Routine postoperative troponin surveillance in non-cardiac surgical patients older than 45 years who require a postoperative night in the hospital is potentially cost effective and should be considered [40]. Therefore, monitoring cardiac biomarkers in elderly patients within 7 days after abdominal surgery is recommended [41]. Patients with electrocardiographic changes or symptoms suggestive of MINS require serial hsTnT measurements, similar to nonsurgical procedures [42]. For countries where hsTnT measurements are not widely available, this predictive model could serve as a screening tool for MINS after major abdominal surgery. Furthermore, among patients who develop MINS, dabigatran has been shown to lower the risk of major vascular complications and could be used as a medication [43].

Several limitations must be considered when interpreting the results. Firstly, during the data collection process, we excluded patients with missing intraoperative fluid replacement volume, blood loss, height, and weight, which may have resulted in the exclusion of patients at risk of potential myocardial injury. Patients without preoperative or postoperative cardiac troponin detection were excluded during patient selection for this study. We acknowledge that missing data inevitably introduce unavoidable biases into our statistical analysis. Excluding patients with missing these important variables may lead to sample bias, thereby affecting the analysis of potential associations and trends. In an international prospective cohort study involving 15,133 patients aged 45 years and older undergoing noncardiac surgery requiring hospitalization, multivariable analysis revealed that peak TnT thresholds of 0.02 ng/mL independently predicted 30-day mortality [44]. High baseline levels of hsTnT may indicate the presence of cardiovascular complications or acute coronary syndrome in patients, which could confound the relationship between predictor variables and the primary outcome (MINS). Excluding patients with preoperative hsTnT levels exceeding 20 ng/L due to identifiable ischemic causes can contribute to ensuring homogeneity within the study population, but may also limit the generalizability, particularly for populations with cardiovascular disease or high baseline levels of cardiac troponin T. Although excluding these patients may underestimate the true incidence of MINS, it ensures a more homogeneous study population and enables a focused evaluation of risk factors relevant to this subgroup. Future studies will further explore the predictive performance of the model in broader patient cohorts to validate its clinical utility in different populations.

Second, retrospective studies are susceptible to inherent biases in patient recruitment and data collection. In this study, postoperative troponin detection was determined by the surgeon. Many factors influence this clinical decision by the surgeon, such as advanced age, preexisting cardiovascular disease, or prolonged surgical duration, typically undergo routine blood sampling for cardiac troponin measurement. Blood samples for troponin measurement may also be drawn when patients experience symptoms. This is inherent to the retrospective nature of the study and may introduce some degree of bias. Our study aims to predict postoperative MINS by identifying risk factors. When patients exhibit high-risk factors included in this study, we recommend that attending/surgical physicians promptly conduct troponin testing postoperatively.

Third, while previous studies have reported that heart rates over 100 bpm for 15 min are associated with MINS, the heart rate elevations were not examined as a factor in our current study due to data availability limitations and the complexity associated with integrating additional variables into our predictive model. The potential significance of this factor deserved to be examined. Besides, we are working on methods to improve it to include surgery urgency in future iterations of the model, which addition would allow for a more nuanced risk assessment, taking into account the unique challenges and risks associated with emergency versus elective surgeries.

Fourth, although we validated our model using external data, the MINS population in the validation cohort was still limited. The relatively small sample size may limit our ability to detect smaller effect sizes or associations within the data. There is an increased risk of Type II errors, where true effects are not identified as statistically significant. Although the sample size of the development cohort (3326 patients) is significantly larger compared to the validation cohort (220 patients), the difference in the AUC of the model between the two cohorts is not substantial. Models trained on large development cohorts may capture a broader range of patient characteristics, clinical scenarios, and variability in outcomes, enhancing their generalizability to diverse patient populations. In contrast, models validated on smaller cohorts may not fully capture the heterogeneity present in the broader patient population. Consequently, the performance of our model needs to be further evaluated in a larger population and in a broader array of settings. Overall, our findings should be interpreted with caution, and future studies with larger and more diverse populations are needed to validate our results.

In conclusion, preoperative hemoglobin levels, preoperative serum ALB levels, infusion volume and blood loss are independent predictors of MINS and are modifiable by clinicians or anesthesiologists. Our predictive model, based on routinely available preoperative information, is a highly accessible tool that may be useful for identifying moderate-to-high risk patients before surgery, allowing for early intervention and individualized perioperative decision-making.

## 5 Conclusions

Preoperative hemoglobin levels, serum ALB concentrations, infusion volumes, and blood loss have been identified as independent predictors of MINS and are subject to modifications by clinicians or anesthesiologists. The predictive model we propose, grounded in routinely available preoperative information, represents a highly accessible tool. It holds the potential to effectively identify patients at moderate-to-high risk before surgery, enabling timely intervention and facilitating personalized perioperative decision-making.

## Implication statement

We developed a predictive model for myocardial injury after major abdominal surgery that facilitates early identification of postoperative myocardial injury and further aids in early intervention and personalized decision-making.

## Funding statement

This research was partly supported by the Medical Scientific Research Foundation of Guangdong Province, China (A2021445, Zhongming Cao).

## Prior conference presentations

Preliminary findings were not presented at any meeting.

## Data availability statement

Data will be available on request. Further inquiries can be directed to the corresponding author.

## CRediT authorship contribution statement

**Guifen Fan:** Writing – original draft, Investigation, Data curation, Conceptualization. **Hanjin Lai:** Writing – review & editing, Validation, Methodology, Investigation, Formal analysis, Conceptualization. **Xiwen Wang:** Writing – review & editing, Visualization, Software, Conceptualization. **Yulu Feng:** Methodology. **Zhongming Cao:** Supervision, Resources, Project administration, Funding acquisition, Data curation, Conceptualization. **Yuxin Qiu:** Supervision, Resources, Investigation, Data curation, Conceptualization. **Shihong Wen:** Writing – review & editing, Supervision, Resources, Project administration, Funding acquisition, Data curation, Conceptualization.

## Declaration of competing interest

The authors declare that they have no known competing financial interests or personal relationships that could have appeared to influence the work reported in this paper.

## List of abbreviations

MINS: Myocardial injury after non-cardiac surgery
AUC: Area under the receiver operating characteristic curve
ASA: American Society of Anesthesiologists
STROBE: Strengthening the Reporting of Observational Studies in Epidemiology
TRIPOD: Transparent Reporting of a Multivariable Prediction Model for Individual Prognosis or Diagnosis
hsTnT: High-sensitivity troponin T
BMI: Body mass index
PLT: Platelet
ACC/AHA: American College of Cardiology and American Heart Association
VIF: Variance inflation factor
ROC: Receiver operating characteristic
CI: Confidence interval
RMA: Robust Multiarray Analysis
IQR: Inter quartile range
OR: Odds ratio
LASSO: Least absolute shrinkage and selection operator
HB: hemoglobin
ALB: Albumin
SAS: Surgical Apgar score
